# Avian haemosporidians of the genera *Plasmodium* and *Haemoproteus* from resident and Neotropical migrant birds in Colombia

**DOI:** 10.1007/s00436-024-08260-8

**Published:** 2024-06-26

**Authors:** Maria Camila Hernández-Ospina, Diego Chitan-Guerrero, Johnathan Alvarez-Londoño, Mauricio Bohada-Murillo, Estefani T. Martínez-Sánchez, Fredy A. Rivera-Páez, Gabriel J. Castaño-Villa

**Affiliations:** 1 Grupo de Investigación en Genética, Biodiversidad y Manejo de Ecosistemas—GEBIOME, Departamento de Ciencias Biológicas, Facultad de Ciencias Exactas y Naturales, Universidad de Caldas, Calle 65 No. 26-10 A.A 275, Manizales, Caldas Colombia; 2 Maestría en Ciencias Biológicas, Facultad de Ciencias Exactas y Naturales, Universidad de Caldas, Calle 65 No. 26-10 A.A 275, Manizales, Caldas Colombia; 3 Grupo de Investigación en Ecosistemas Tropicales, Departamento de Ciencias Biológicas, Facultad de Ciencias Exactas y Naturales, Universidad de Caldas, Calle 65 No. 26-10 A.A 275, Manizales, Caldas Colombia; 4 Doctorado en Ciencias-Biología, Facultad de Ciencias Exactas y Naturales, Universidad de Caldas, Calle 65 No. 26-10 A.A 275, Manizales, Caldas Colombia; 5 Grupo de Investigación en Genética, Biodiversidad y Manejo de Ecosistemas—GEBIOME, Departamento de Desarrollo Rural y Recursos Naturales, Facultad de Ciencias Agropecuarias, Universidad de Caldas, Carrera 35 No. 62-160 A.A 275, Manizales, Caldas Colombia

**Keywords:** Avian malaria, Blood, Farmlands, Hummingbirds, Secondary forest, Thraupidae

## Abstract

Avian haemosporidians of the genera *Plasmodium* and *Haemoproteus* are a group of widely distributed blood parasites that can negatively affect the fitness of their hosts. Colombia contains the greatest diversity of birds on the planet, but knowledge about the associations between haemosporidian and its avifauna is scarce and fragmented. We collected blood samples from 255 birds (203 residents and 52 neotropical migrants) belonging to 27 families and 108 species. The study was conducted in six localities in the inter-Andean valleys of the Cauca and Magdalena rivers. Parasites of the genera *Plasmodium* and *Haemoproteus* were identified in the samples by morphological and molecular analysis of a fragment of the mitochondrial gene *cyt b*. Among the samples, 9.3% (*n* = 24) were positive for *Plasmodium* or *Haemoproteus*. Co-infection with *Plasmodium* and *Haemoproteus* was found in Red-eyed Vireo﻿. Seventeen haemosporidian lineages were identified, five of which were reported for the first time in resident birds (Common Ground Dove, Checker-throated Stipplethroat, Tropical Kingbird, Pale-breasted Thrush, and Ruddy-breasted Seedeater) and one in the Summer Tanager (neotropical migrant). The research results confirm the wide diversity of haemosporidian present in tropical lowlands and the possible role of neotropical migratory birds in dissemination on haemosporidian along their migratory routes.

**Supplementary Information:**

The online version contains supplementary material available at 10.1007/s00436-024-08260-8.

## Introduction

Haemosporidian (Apicomplexa: Haemosporida) are a group of hemoparasites that infect a wide range of vertebrate hosts globally, including reptiles, amphibians, mammals, and birds (Ricklefs and Fallon 2002; Valkiūnas 2005; Lacorte et al. 2013; Ellis et al. 2020). Birds are susceptible to infection by mosquito-borne *Plasmodium* genera (Culicidae) and *Haemoproteus* (subgenera *Parahaemoproteus* and *Haemoproteus*), transmitted by Ceratopogonidae and Hippoboscidae flies, respectively (Valkiūnas 2005; Santiago-Alarcon et al. 2012; Toscani Field et al. 2018). Haemosporidians of the genera *Plasmodium* and *Haemoproteus* have complex life cycles with sexual reproductive stages in arthropod vectors and asexual reproduction in their avian hosts (Valkiūnas 2005). However, asexual reproduction may be disrupted in a non-competent avian host, and the mature forms of the parasite (gametocytes), which are infectious to the vector, are not generated (i.e., abortive infection) (Valkiūnas 2005; Valkiūnas and Iezhova 2017). Infections caused by haemosporidian can have adverse effects on birds’ reproductive success and survival, due to the damage caused in tissues and organs such as the spleen, liver, and brain, and consequently, affect their populations (Palinauskas et al. 2008, 2015; Risely et al. 2018).

Over the past few decades, the implementation of morphological and molecular analyses has allowed the identification of a wide diversity of avian haemosporidian morphospecies and lineages in the Neotropical region (Bensch et al. 2009; González et al. 2015; Fecchio et al. 2018; 2019; Ellis et al. 2020). In the neotropics, there have been reports of resident and migratory birds infected by the genera *Plasmodium* and *Haemoproteus*, which could play a significant role in the dispersal of haemosporidian throughout the Americas (Valkiūnas 2005; DeBrock et al. 2021; Lotta-Arévalo et al. 2023). In Colombia, 140 neotropical migratory bird species are recorded during the spring and fall migrations (Echeverry-Galvis et al. 2022). Infection by *Plasmodium* (*Haemamoeba*) *cathemerium*, *Haemoproteus* (*Parahaemoproteus*) *vireonis*, or *Haemoproteus* (*Parahaemoproteus*) *coatneyi* has been detected in birds of the Vireonidae, Turdidae, or Cardinalidae families (González et al. 2015; Pulgarín-R et al. 2019). In Colombia, there is a limited and fragmented knowledge about haemosporidians and their interaction with wild birds, with studies mainly conducted in localities of the Caribbean, Orinoco, and High Andean regions (González et al. 2015; Alvarez-Londoño et al. 2022; Lotta-Arévalo et al. 2023). This lack of information hinders the understanding of birds’ role in the epidemiology of *Plasmodium* and *Haemoproteus*.

In this context, and considering that the regions encompassing the inter-Andean valleys of the Cauca and Magdalena rivers are include in the migration routes of at least 83 species of neotropical migratory birds (Fierro-Calderón and Eusse 2010; Naranjo et al. 2012), this region could serve as a “transmission zone” where resident-migratory bird species and haemosporidians of South and North American origin converge (Moens and Pérez-Tris 2016; Fecchio et al. 2018). This research aimed to determine the diversity and prevalence of *Plasmodium* and *Haemoproteus* in neotropical resident and migratory wild birds at six locations in the inter-Andean valleys of the Cauca and Magdalena rivers.

## Materials and methods

### Study area

**Fig. 1:**
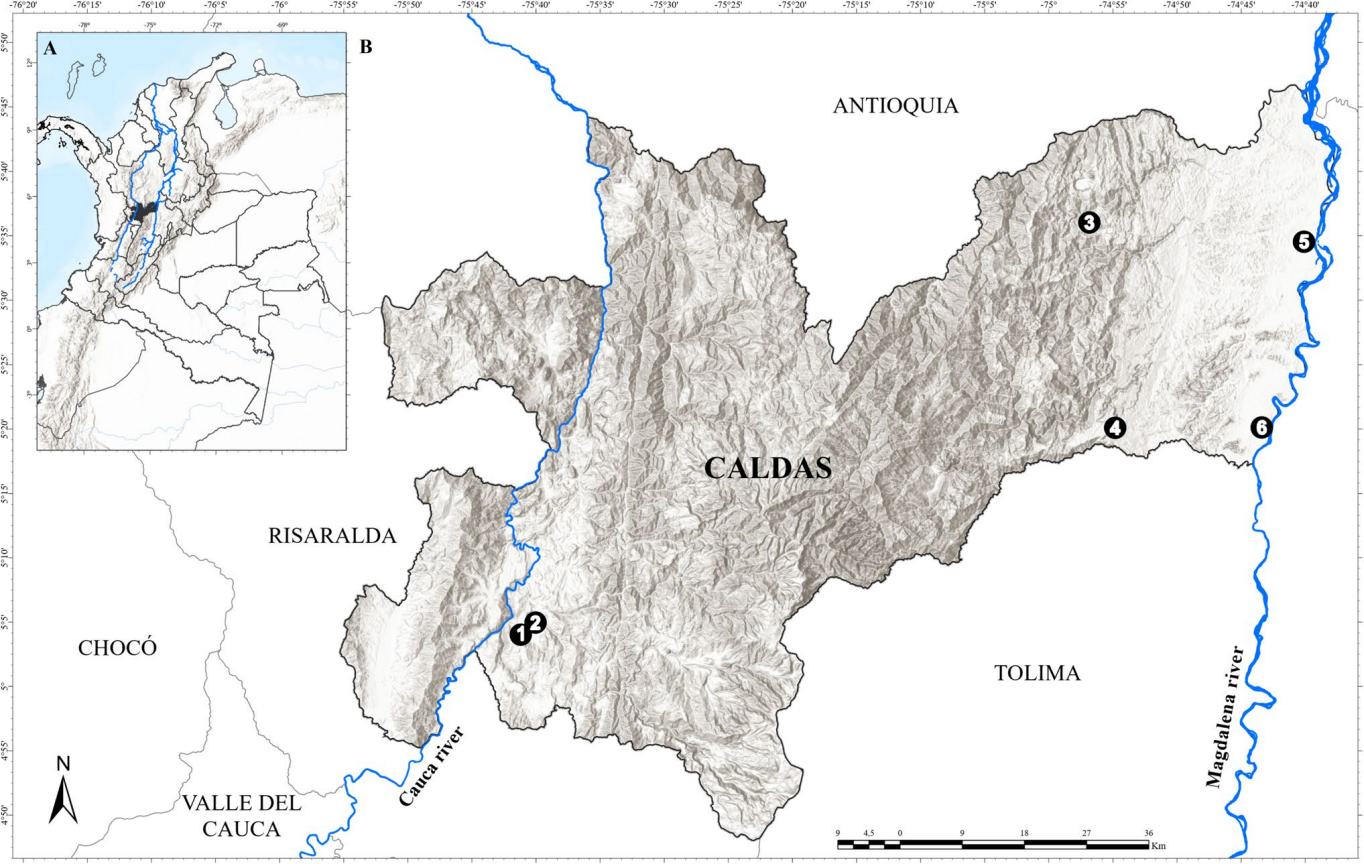
Study area. **A** Map of Colombia depicting department of Caldas (black). **B** Sampled localities in Cauca River Valley, (**1**) Granja Luker and (**2**) Granja Montelindo; sampled localities in Magdalena River Valley, (**3**) Corregimiento Berlín, (**4**) Finca El Edén, (**5**) Hacienda Alcaparrosa, and (**6**) Charca de Guarinocito

The study was conducted in six localities within the department of Caldas, Colombia (Latitude: 4.07194, Longitude: − 75.95722). The localities are situated in the inter-Andean valleys of the Cauca (Municipality of Palestina) and Magdalena rivers (Municipalities of La Dorada, Samaná and Victoria) (Fig. 1). The six localities are found within an elevation range of 126 and 1050 m a.s.l. They experience a bimodal rainfall pattern (average annual precipitation ranging from 2000 to 4000 mm). The highest rainfall occurs between March and June and between September and December. The average temperature ranges between 20 and 27 °C (Martínez-Sánchez et al. 2018). The sampled localities in the inter-Andean valley of the Magdalena River are farms dedicated to the production of avocado (*Persea americana* Mill.) or cocoa (*Theobroma cacao* L.), or buffalo rearing (*Bubalus bubalis* L.) and two secondary forests. The two locations that were sampled in the inter-Andean valley of the Cauca River correspond to two farms that produce cocoa (*T. cacao*) or citrus (*Citrus* spp.) production (Table 1).

**Table 1:** Descriptions of the localities in the department of Caldas where birds were sampled, Colombia. The localities correspond to the numbers presented in Fig. 1

Municipality	Locality	Locality number	Latitude	Longitude	Elevation (m above sea level)	Habitat type
Palestina	Granja Luker	1	5.0735	− 75.682	1050	Cocoa agroforestry systems (*Theobroma cacao* L.)
	Granja Montelindo	2	5.0753	− 75.6730	1010	Monocrop (*Citrus nobilis* Lour.)
Samaná	Corregimiento Berlín	3	5.6005	− 74.9473	791	Secondary forest
Victoria	Finca El Edén	4	5.3348	− 74.9137	1000	Mixed-cropping (*Persea americana* M. and *Theobroma cacao* L.)
La Dorada	Hacienda Alcaparrosa	5	5.5756	− 74.6683	126	Grazing pasture with weeds and dispersed tree
	Charca de Guarinocito	6	5.3353	− 74.7232	208	Secondary forest

### Collection and processing of bird blood samples

Wild birds were captured from September 2021 to February 2022 using six mist nets (12 × 2.5 m × 36 mm) at each location for five consecutive days, with a total sampling intensity of 1980 h/net. The nets were operated between 06:00 and 17:00 h. Captured birds were marked with a small cut on the first rectrix to avoid recounts and were subsequently released at the location where they were captured (Martínez-Sánchez et al. 2018). The bird taxonomy followed the nomenclature of Remsen et al. (2023). Residency status (neotropical migratory or resident) was determined according to Echeverry-Galvis et al. (2022). A blood sample (approximately 20 to 50 μl) was obtained from each captured bird through brachial vein puncture using 25G and 27G gauge needles (Busi et al. 2020). To obtain the hummingbird blood sample, a small cut was made near the fingernail (Owen 2011). The samples were used to create blood smears (at least two per bird), fixed in absolute methanol for 5 min, and stained with 5% GIEMSA solution, pH 7.2 for 45 min (Alvarez-Londoño et al. 2022). The remaining blood was also deposited on FTA cards (*Flinders Technology Associates*) for subsequent molecular analysis.

### Morphological analysis

For morphological identification of haemosporidians, blood smears were analyzed using a light microscope, specifically an Olympus BX43 optical microscope, with 40 × and 100 × objectives; approximately 100–150 fields were visualized per objective (Valkiūnas 2005). The parasites were identified using specialized taxonomic guides (Valkiūnas 2005; Valkiūnas and Iezhova 2018; 2022). Images of the parasites were captured using an Olympus DP28 digital camera and edited with Olympus CellSens Standard v3.22.11 software.

### Molecular analysis

DNA extraction from blood samples stored on FTA cards was performed using the Wizard Genomic DNA Purification kit (Promega Corporation, Madison, USA) following the manufacturer’s instructions*.* To detect *Plasmodium* and *Haemoproteus* DNA, nested Polymerase Chain Reaction (PCR) testing was conducted on a fragment of a cytochrome *b* (*cyt b*) gene. The initial PCR utilized primers AE064/AE066 to amplify a 1109 bp fragment for all three genera of haemosporidians (*Plasmodium*, *Haemoproteus*, and *Leucocytozoon*) (Pacheco et al. 2018). One microliter of the initial PCR was utilized for the second PCR, utilizing the HaemF/HaemR2 primers that amplify a 480 bp fragment for *Plasmodium* and *Haemoproteus* (Hellgren et al. 2004). To determine *Plasmodium* DNA, a nested PCR was conducted using the initial PCR amplicon and primers AE983/AE985 which amplify a 580 bp fragment (Pacheco et al. 2018). Similarly, a nested PCR was performed using the initial PCR amplicon and primers AE980/AE982 to determine *Haemoproteus* DNA, resulting in amplification of a 346 bp fragment (Pacheco et al. 2018). Samples positive by PCR with primers AE983/AE985 and primers AE980/AE982 were considered coinfections. All PCR reactions were conducted with both a negative control (H_2_Odd) and positive control (*Haemoproteus columbae*) (Alvarez-Londoño et al. 2022). To visualize PCR products, horizontal electrophoresis was carried out using 1% agarose gels with 1X TBE buffer, stained with SYBR SAFE (Thermo Fisher Scientific, Waltham, USA), and visualized in UV photodocumenter. Positive samples for HaemF/HaemR2 were sent to Macrogen (Seoul, South Korea) for purification and Sanger sequencing. The obtained sequences were edited in the Geneious Prime program (2023.0.4. https://www.geneious.com/) and aligned in the MEGA11 software (Tamura et al. 2021). To identify *Plasmodium* and *Haemoproteus* genetic lineages, we compared our sequences with sequences deposited in the public databases MalAvi (http://130.235.244.92/Malavi/, Bensch et al. 2009) and GenBank (https://blast.ncbi.nlm.nih.gov/Blast.cgi). Sequences that had at least one nucleotide difference with the MalAvi sequences were considered new lineages and were named according to the nomenclature proposed by Bensch et al. (2009).

Phylogenetic reconstruction was conducted using Bayesian inference on the identified lineages of *Plasmodium* and *Haemoproteus*. The alignments included our sequences and sequences of the *cyt b* gene obtained from the MalAvi database which were linked to morphospecies, and a sequence of *Leucocytozoon buteonis* BUTREG01 [DQ177264] was used as an outgroup. The total length of the alignments was 480 bp. The General Time Reversible model with invariant sites and gamma distribution (GTR + I + G) was selected according to the corrected Akaike information criterion using jModeltest v.2.1.6 (Darriba et al. 2012). Bayesian analysis was carried out using MrBayes v3.2.7a (Ronquist and Huelsenbeck 2003) via the CIPRESS Science Gateway v3.3 (Miller et al. 2010). Two independent Markov and Monte Carlo chains (MCMC) were used simultaneously for a total of 15 million generations, with four chains sampled every 1000 generations. Convergence was evaluated by calculating the mean and standard deviation of the frequencies divided between the two runs below 0.01 and graphically using Tracer (Rambaut and Drummond 2007). A total of 25% of the trees were discarded as run periods, and the remaining trees were used to create a consensus tree applying a 50% majority rule. The phylogeny was visualized using FigTree v1.3.1 (Rambaut 2013). All obtained sequences were deposited in MalAvi and GenBank. The percent prevalence of *Plasmodium* or *Haemoproteus* in birds was determined using the following equation [(number of infected individuals / number of individuals examined) × 100] (Bush et al. 1997). Molecularly positive samples that did not present forms of the parasite in the blood were not included in the prevalence estimation (Alvarez-Londoño et al. 2022).

## Results

**Fig. 2:**
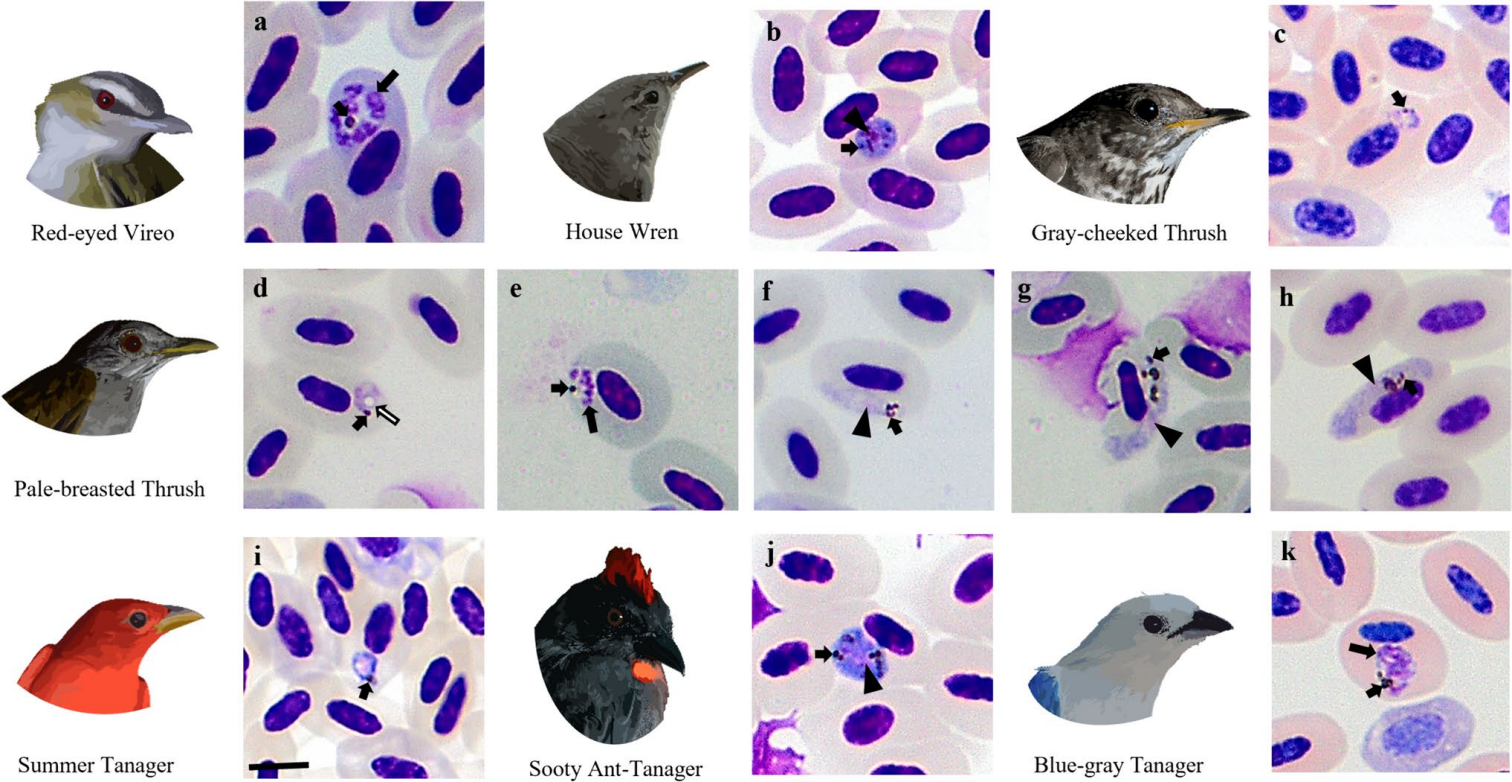
Parasites of the genera *Plasmodium* found in the study. **a** Erythrocytic meront of *Plasmodium* sp. (VIOLI03) in the Red-eyed Vireo. **b** Macrogametocyte of *Plasmodium* sp. (SETAUD23) in the House Wren. **c** Trophozoite of *Plasmodium* (*H*.) *matutinum* (LINN1) in the Gray-cheeked Thrush. **d** Trophozoite of *Plasmodium unalis* (TULEU09) in the Pale-breasted Thrush. **e** Erythrocytic meront of *Plasmodium unalis* (TULEU09) in the Pale-breasted Thrush. **f** Microgametocyte of *Plasmodium unalis* (TULEU09) in the Pale-breasted Thrush. **g–h** Macrogametocyte of *Plasmodium unalis* (TULEU09) in the Pale-breasted Thrush. **i** Trophozoite of *Plasmodium* sp. (PIRUB04) in the Summer Tanager. **j** Macrogametocyte of *Plasmodium* sp. (EMBHER01) in the Sooty Ant-Tanager. **k** Young erythrocytic meront of *Plasmodium* sp. (PADOM09) in the Blue-gray Tanager. *Short black arrow* pigment granules. *Black long arrow* merozoite. *Black arrowhead* parasite nucleus. *White long arrow* vacuole. Scale bar = 10 μm

**Fig. 3:**
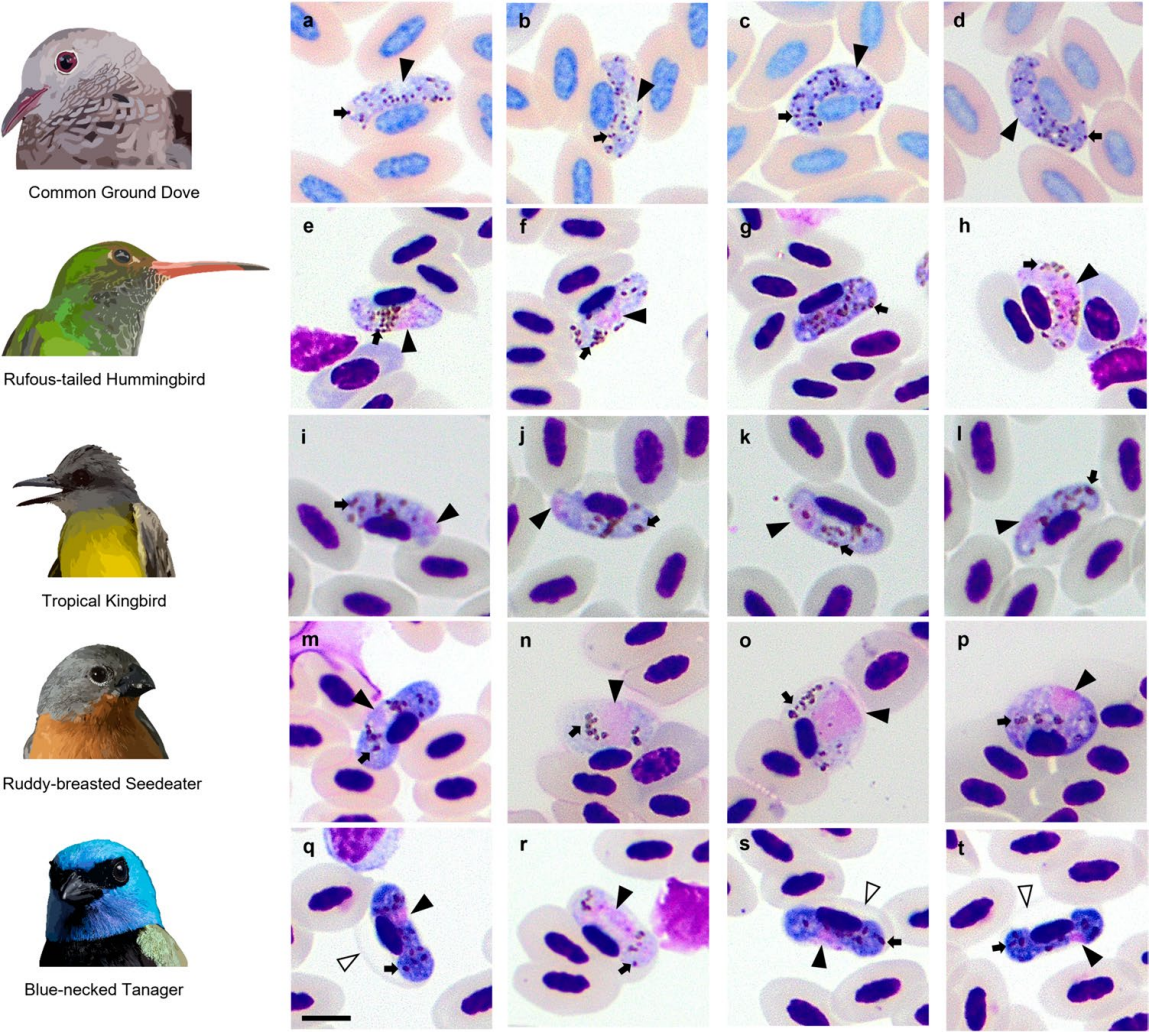
Parasites of the genera *Haemoproteus* found in the study. **a–d** Macrogametocytes of *H*. (*H*.) *paramultipigmentatus* in the Common Ground Dove. **e, f, h** Microgametocytes of *H*. (*P*.) *witti* in the Rufous-tailed Hummingbird. **g** Macrogametocyte of *H*. (*P*.) *witti* in Rufous-tailed Hummingbird. **i–l** Macrogametocytes of *H*. (*P*.) *tyranni* in the Tropical Kingbird. **m, p** Macrogametocytes of *H*. (*P*.) *nucleocentralis* in the Ruddy-breasted Seedeater. **n, o** Microgametocytes of *H*. (*P*.) *nucleocentralis* in the Ruddy-breasted Seedeater. **q, s, t** Macrogametocytes of *H*. (*P*.) *erythrogravidus* in the Blue-necked Tanager. **r** Microgametocyte of *H*. (*P*.) *erythrogravidus* in the Blue-necked Tanager. *Black arrowhead* parasite nucleus. *Short black arrow* pigment granules. *White arrowhead* protrusions of infected erythrocyte envelope. Scale bar = 10 μm

A total of 255 birds belonging to 108 species and 27 families (79% resident, 20% neotropical migrants, and 1% introduced) were examined (Tables 2 and 3). Twelve percent of the bird species are neotropical migrants of Tyrannidae, Vireonidae, Turdidae, Parulidae, and Cardinalidae (Tables 2 and 3). The 68.5% of the examined birds were captured in agricultural areas, while 31.5% were found in secondary forests. Parasites from the genera *Plasmodium* and *Haemoproteus* were identified in 20 passerine and non-passerine species (Table 2). The prevalence of *Plasmodium* was 4.3% (11/255), while the prevalence of *Haemoproteus* was 3.5% (9/255). Seventy-one percent of the haemosporidian-infected birds were residents, while 29% were neotropical migrants. Neotropical migratory birds Red-eyed Vireo (*Vireo olivaceus*), Gray-cheeked Thrush (*Catharus minimus*), Swainson’s Thrush (*Catharus ustulatus*), and Northern Waterthrush (*Parkesia noveboracensis*) were found infected by haemosporidians. These birds were captured during the fall migration (between October and November 2021) (Table 2). At the same time, the Summer Tanager (*Piranga rubra*) and Scarlet Tanager (*Piranga olivacea*) were captured in winter (February 2022) (Table 2). Morphologically, *Plasmodium* (*Novyella*) *unalis* infection (trophozoites, meronts, and gametocytes) was identified in the Pale-breasted Thrush (*Turdus leucomelas*) (Fig. 2) and *Haemoproteus* (*Haemoproteus*) *paramultipigmentatus* infection in the Common Ground Dove (*Columbina passerina*), *Haemoproteus* (*Parahaemoproteus*) *witti* in the Rufous-tailed Hummingbird (*Amazilia tzacatl*), *Haemoproteus* (*Parahaemoproteus*) *nucleocentralis* in the Ruddy-breasted Seedeater (*Sporophila minuta*), and *Haemoproteus* (*Parahaemoproteus*) *erythrogravidus* in the Blue-necked Tanager (*Stilpnia cyanicollis*) in the Magdalena Valley (Fig. 3). *Haemoproteus* (*Parahaemoproteus*) *tyranni* was detected in a Tropical Kingbird (*Tyrannus melancholicus*) in the Cauca River Valley (Table 2; Fig. 3).

**Table 2:** Avian hosts positive with *Plasmodium* spp. and *Haemoproteus* spp. in the department of Caldas. The GenBank accession codes in bold correspond to the sequences obtained in this study. Localities where birds were sampled in the department of Caldas, Colombia. The localities correspond to the numbers presented in Fig. 1

Order/family	Host species English name (Scientific name)	No. infected birds/no. examined birds (% prevalence)	Parasite species and lineages from MalAvi (% similarity)	Number of birds by locality [no. bird infected]					
				1	2	3	4	5	6
Columbiformes/									
Columbidae	Common ground dove (*Columbina passerina*)	1/2 (50)	*Haemoproteus* (*H*.) *paramultipigmentatus* COLPAS11*				2[1]		
	Ruddy ground dove (*Columbina talpacoti*)	2/4 (50)	*Haemoproteus* sp.^**b**^		4[2]				
Apodiformes/									
Trochilidae	Rufous-tailed hummingbird (*Amazilia tzacatl*)	1/2 (50)	*Haemoproteus* (*P*.) *witti ***EUPMAC01** (100%)		2[1]				
Passeriformes/									
Thamnophilidae	Checker-throated stipplethroath (*Epinecrophylla fulviventris*)	1/4 (25)	*Plasmodium* sp. EPIFUL01*			4[1]			
Formicariidae	Black-faced antthrush (*Formicarius analis*)	1/1 (100)	*Plasmodium* sp.^**S**^			1[1]			
Tyrannidae	Tropical kingbird (*Tyrannus melancholicus*)	2/2 (100)	*Haemoproteus* (*P*.) *tyranni ***MYMAC03** (100%)		2[2]				
	Tropical kingbird (*Tyrannus melancholicus*)		*Haemoproteus* (*P*.) *tyranni* TYRMEL03*						
Vireonidae	Red-eyed vireo (*Vireo olivaceus*)**ˆ**	1/1 (100)	*Plasmodium* sp. **VIOLI03** (100%)	1[1]					
			*Haemoproteus* sp.^**S**^						
Troglodytidae	House wren (*Troglodytes aedon*)	1/3 (33.3)	*Plasmodium* sp. **SETAUD23** (100%)	1[1]			2		
Turdidae	Gray-cheeked thrush (*Catharus minimus*)**ˆ**	1/4 (25)	*Plasmodium* (*H*.) *matutinum* **LINN1** (100%)			1		1[1]	2
	Swainson’s thrush (*Catharus ustulatus*)**ˆ**	1/22 (4.54)	*Plasmodium* sp. **BT7** (100%)	2		7	7	5[1]	1
	Pale-breasted thrush (*Turdus leucomelas*)	1/1 (100)	*Plasmodium unalis* TULEU09*					1[1]	
Passerellidae	Orange-billed sparrow (*Arremon aurantiirostris*)	1/1 (100)	*Haemoproteus* sp. **ATLBRU01** (100%)			1[1]			
Parulidae	Northern waterthrush (*Parkesia noveboracensis*)**ˆ**	1/4 (25)	*Plasmodium* sp.^**a, b**^	2[1]				1	1
Cardinalidae	Summer tanager (*Piranga rubra*)**ˆ**	1/1 (100)	*Plasmodium* sp. PIRUB04*				1[1]		
	Scarlet tanager (*Piranga olivacea*)**ˆ**	1/1 (100)	*Plasmodium* sp. **TACTHA01**^**a**^** (**100%)				1[1]		
	Sooty ant-tanager (*Habia gutturalis*)	3/5 (60)	*Plasmodium* sp. **EMBHER01** (100%)			5[3]			
Thraupidae	Ruddy-breasted seedeater (*Sporophila minuta*)	1/6 (14)	*Haemoproteus* (*P*.) *nucleocentralis* SPOMIN01*				6[1]		
	Bananaquit (*Coereba flaveola*)	1/3 (33.3)	*Plasmodium* sp. **PADOM09** (100%)	1[1]		1	1		
	Blue-necked tanager (*Stilpnia cyanicollis*)	1/1 (100)	*Haemoproteus* (*P*.) *erythrogravidus* **TANCYA01** (100%)				1[1]		
	Blue-gray tanager (*Thraupis episcopus*)	1/4 (25)	*Plasmodium* sp. **PADOM09** (100%)		4[1]				

*New haemosporidian lineages reported in the study

**ˆ**Neotropical migrant bird

^**S**^Presence of blood forms, PCR negative

^**a**^Molecularly positive samples that did not present forms of the parasite in the blood

^**b**^Unidentified lineage (low quality sequence)

**Table 3:** Non-infected bird species by haemosporidian parasites caught in the department of Caldas—Colombia (the localities correspond to the numbers presented in Fig. 1)

Order	Family	Bird species	No. of examined bird	Number of birds by locality					
				1	2	3	4	5	6
Columbiformes	Columbidae	*Leptotila verreauxi*	2					1	1
Apodiformes	Trochilidae	*Glaucis hirsutus*	1			1			
		*Threnetes ruckeri*	1			1			
		*Phaethornis striigularis*	1			1			
		*Phaethornis anthophilus*	2			2			
		*Phaethornis guy*	2			2			
		*Chlorostilbon gibsoni*	1					1	
		*Chalybura buffonii*	6			1	5		
		*Polyerata amabilis*	1				1		
Charadriiformes	Charadriidae	*Vanellus chilensis*	1		1				
Coraciformes	Momotidae	*Hylomanes momotula*	1			1			
		*Momotus subrufescens*	2					2	
	Alcedinidae	*Chloroceryle aenea*	2					1	1
Galbuliformes	Bucconidae	*Notharchus tectus*	2					2	
		*Hypnelus ruficollis*	1						1
Piciformes	Ramphastidae	*Pteroglossus torquatus*	1				1		
	Picidae	*Melanerpes rubricapillus*	2	1			1		
		*Colaptes punctigula*	1	1					
Psittaciformes	Psittacidae	*Forpus conspicillatus*	3	1			2		
Passeriformes	Thamnophilidae	*Cercomacra nigricans*	2	2					
		*Sipia palliata*	1			1			
		*Hafferia immaculata*	2			2			
		*Gymnopithys bicolor*	3			3			
	Furnariidae	*Dendrocincla fuliginosa*	8	3		4		1	
		*Glyphorynchus spirurus*	1			1			
		*Xiphorhynchus triangularis*	2					2	
		*Lepidocolaptes souleyetii*	1	1					
		*Furnarius leucopus*	4					3	1
		*Certhiaxis cinnamomeus*	1						1
		*Synallaxis albescens*	1				1		
	Pipridae	*Lepidothrix coronata*	3			3			
		*Manacus manacus*	5			4		1	
		*Machaeropterus striolatus*	3			3			
		*Ceratopipra erythrocephala*	1				1		
	Tityridae	*Pachyramphus cinnamomeus*	1			1			
	Tyrannidae	*Mionectes oleagineus*	7	1	2	4			
		*Leptopogon amaurocephalus*	3			3			
		*Tolmomyias sulphurescens*	1	1					
		*Oncostoma olivaceum*	2			2			
		*Poecilotriccus sylvia*	2	2					
		*Todirostrum cinereum*	3	1			2		
		*Zimmerius chrysops*	1		1				
		*Camptostoma obsoletum*	1				1		
		*Elaenia flavogaster*	1				1		
		*Elaenia chiriquensis*	1				1		
		*Tyrannulus elatus*	1				1		
		*Phyllomyias griseiceps*	1				1		
		*Legatus leucophaius*	1				1		
		*Pitangus sulphuratus*	1	1					
		*Phaeomyias murina*	1	1					
		*Myiodynastes maculatus*	1		1				
		*Myiozetetes cayanensis*	3				1	1	1
		*Myiozetetes similis*	1					1	
		*Myiarchus panamensis*	1			1			
		Empidonax virescens*	1			1			
		Empidonax traillii*	1	1					
		*Contopus cinereus*	1		1				
	Vireonidae	*Vireo leucophrys*	1	1					
	Hirundinidae	*Stelgidopteryx ruficollis*	2	1	1				
	Troglodytidae	*Microcerculus marginatus*	1			1			
	Turdidae	*Turdus grayi*	1		1				
		*Turdus ignobilis*	2			2			
	Estrildidae	*Lonchura malacca*^**+**^	1		1				
	Fringillidae	*Euphonia laniirostris*	1	1					
	Icteridae	*Icterus nigrogularis*	1		1				
	Parulidae	Leiothlypis peregrina*	1				1		
		Geothlypis philadelphia*	1	1					
		Setophaga castanea*	7				6		1
		Setophaga ruticilla*	1	1					
		Setophaga petechia*	7	5	2				
		*Basileuterus rufifrons*	1				1		
	Mitrospingidae	*Mitrospingus cassinii*	1			1			
	Cardinalidae	*Cyanoloxia brissonii*	2	2					
	Thraupidae	*Sicalis flaveola*	6		3		3		
		*Volatinia jacarina*	12	2	2		8		
		*Eucometis penicillata*	3			2		1	
		*Ramphocelus dimidiatus*	5					5	
		*Dacnis lineata*	1				1		
		*Dacnis cayana*	1				1		
		*Sporophila crassirostris*	1	1					
		*Sporophila intermedia*	3	1			2		
		*Sporophila nigricollis*	5	1	3		1		
		*Sporophila schistacea*	2			1	1		
		*Saltator maximus*	1			1			
		*Saltator striatipectus*	1	1					
		*Stilpnia vitriolina*	1				1		
		*Tangara inornata*	1				1		
		*Thraupis palmarum*	1				1		
Total			**184**	**35**	**20**	**50**	**49**	**23**	**7**

*Neotropical migrant bird

^+^Introduced bird

**Fig. 4:**
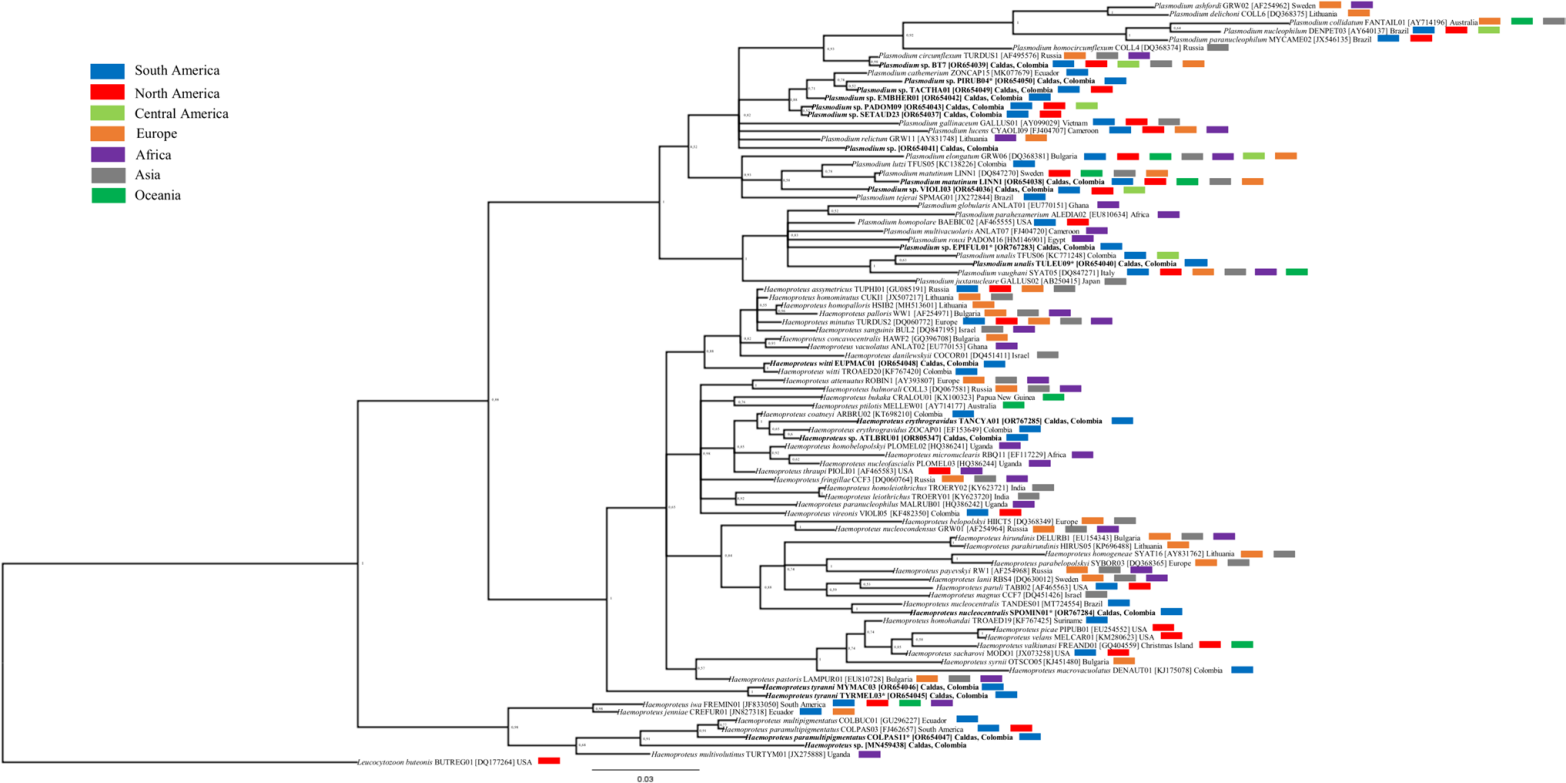
Bayesian phylogeny of *Haemoproteus* spp. and *Plasmodium* spp. based on 117 lineages of cytochrome *b* gene. Morphological species names followed by MalAvi lineage, GenBank accession number within square brackets, and the country where the sequences are reported. The colored rectangles correspond to the geographic regions where the lineages from the MalAvi database have been found. Lineages and sequences reported in this study are shown in bold. The sequence of *Leucocytozoon buteonis* BUTREG01 was used as an outgroup. The scale bar represents the number of nucleotide substitutions per site. Nodal support values indicate posterior probabilities

Molecularly, 17 haemosporidian lineages were determined (10 *Plasmodium* and seven *Haemoproteus*). Lineages that were previously reported in the MalAvi database of *Plasmodium* were recorded in the Red-eyed Vireo (VIOLI03), the House Wren (*Troglodytes aedon*) (SETAUD23), the Gray-cheeked Thrush (LINN1), the Swainson’s Thrush (BT7), the Scarlet Tanager (TACTHA01), the Sooty Ant-Tanager (*Habia gutturalis*) (EMBHER01), the Bananaquit (*Coereba flaveola*) (PADOM09), and the Blue-gray Tanager (*Thraupis episcopus*) (PADOM09)*.* However, *Haemoproteus* lineages were detected in the Rufous-tailed Hummingbird (EUPMAC01), the Tropical Kingbird (MYMAC03), the Orange-billed Sparrow (*Arremon aurantiirostris*) (ATLBRU01), and the Blue-necked Tanager (TANCYA01) (Table 2; Fig. 4)*.* Some of these lineages were documented for the first time in Colombia within resident or neotropical migratory birds (SETAUD23, TACTHA01, VIOLI03, EMBHER01, EUPMAC01, ATLBRU01, TANCYA01, and LINN1). Three new *Plasmodium* lineages were detected, PIRUB04 infecting the Summer Tanager, TULEU09 infecting the Pale-breasted Thrush, and EPIFUL01 infecting the Checker-throated Stipplethroat (*Epinecrophylla fulviventris*). Additionally, three new *Haemoproteus* lineages were found, COLPAS11 infecting the Common Ground Dove, TYRMEL03 infecting the Tropical Kingbird, and SPOMIN01 infecting the Ruddy-breasted Seedeater (Table 2; Fig. 4). Notably, the new *H*. (*P*.) *nucleocentralis* lineage SPOMIN01 was closely related (posterior probability = 1) to the TANDES01 lineage, with an evolutionary distance of 2.4% (Table S1, Fig. 4). The GenBank accession codes obtained for the *cyt b* gene in this study are [OR654036–OR654050, OR767283–OR767285, OR805347]. Likewise, the sequences of the new lineages were deposited in the MalAvi database.

However, no parasitic forms of *Plasmodium* were found in blood smears of the Northern Waterthrush, the Scarlet Tanager, and the Gray-headed Tanager (*Eucometis penicillata*) (Table 2). Only one individual of the Red-eyed Vireo was co-infected with *Plasmodium* sp. (VIOLI03) and *Haemoproteus* (*P*.) sp. (Table 2). The blood smears obtained in this study are located in the Genetics Laboratory of the Universidad de Caldas.

## Discussion

The prevalence of *Plasmodium* spp. or *Haemoproteus* spp. was similar to that established in other localities in Colombia (Rodríguez and Matta 2001; Valkiūnas et al. 2003; Basto et al. 2006; Alvarez-Londoño et al. 2022). It has been suggested that the prevalence of these haemosporidians in birds seems to be determined by habitat characteristics (e.g., lentic water bodies, ponds, or forest type) that support the development, abundance, and diversity of their vectors or by ecophysiological traits of their hosts (González et al. 2015; Alvarez-Londoño et al. 2022). Particularly, one of the locations with the highest number of *Plasmodium* spp. infected birds was the Granja Luker. On this farm, pools are formed as a result of the crop drainage network, and there are bromeliads attached to trees that serve as habitat for mosquitoes of the genus *Wyeomyia*, recognized vectors of avian *Plasmodium* (Bensch et al. 2009; Morcillo et al. 2023).

Seventy percent of the *Plasmodium* lineages reported in our study have previously been detected in America, Europe, Asia, or Australia (Bensch et al. 2009; Fig. 4). However, some of these *Plasmodium* lineages were documented for the first time in Colombia within resident or neotropical migratory birds. In relation to this, we consider it as an extension of the geographic distribution range across the Americas for the SETAUD23 and TACTHA01 lineages of *Plasmodium* sp. detected in House Wren and Scarlet Tanager, respectively. Both lineages have been previously reported in North America in neotropical migratory birds wintering in Colombia (Bensch et al. 2009; Fig. 4). We recorded the geographic expansion in the South American distribution of the VIOLI03 and EMBHER01 lineages of *Plasmodium* sp., as well as the EUPMAC01 lineages of *H.* (*P*.) *witti*, ATLBRU01 of *Haemoproteus* sp., TANCYA01 of *H.* (*P*.) *erythrogravidus*. These lineages had previously been recorded only in other countries of the Americas, such as the USA, Guyana, Peru, or Brazil, in both passerine and non-passerine birds (Beadell et al. 2006; Durrant et al. 2006; Bensch et al. 2009; Fecchio et al. 2019). Likewise, we report the presence of *P*. (*H*.) *matutinum* LINN1 in Colombia infected to a migratory Gray-cheeked Thrush. This *Plasmodium* morphospecies has previously been recorded in North America, Europe, Asia, and Australia, infecting birds of various orders including Gruiformes, Charadriiformes, Strigiformes, or Passeriformes (Bensch et al. 2009; Fig. 4). In particular, *P*. (*H*.) *matutinum* has been observed in birds on the east coast of the USA (states of Michigan and New York) (Bensch et al. 2009). These localities are part of the migration routes of the Gray-cheeked Thrush from their breeding grounds in Asia (northeastern Siberia) and North America (from Alaska to the East Coast) (Udvardy 1994). This suggests that neotropical migratory birds may play a key role in the dispersal of parasites of the genus *Plasmodium* between temperate and tropical regions of the Americas (Hellgren et al. 2007; Fecchio et al. 2018). We reported six new lineages of haemosporidians infecting resident and migratory birds, of which three lineages of *Haemoproteus* (COLPAS11, TYRMEL03, and SPOMIN01) and one lineage of *Plasmodium* (TULEU09) were related to a morphospecies through morphological analysis. The TULEU09 lineage of *P.* (*N*.) *unalis* recorded in the Pale-breasted Thrush appears to be related to the TFUS06 lineage of *P.* (*N*.) *unalis* that has been previously documented in the Great Thrush (*Turdus fuscater*) in Colombia, as well as four other species of the genus *Turdus* in the Atlantic forest of Brazil (*Turdus rufiventris*, *T*. *leucomelas*, *Turdus albicollis*, and *Turdus flavipes*) (Mantilla et al. 2013; Tostes et al. 2018; Fig. 4). In this regard, it has been suggested that there is a high degree of intraspecific polymorphism, and a wide diversity of host species are present within this parasite species (Mantilla et al. 2013; Vanstreels et al. 2015; Tostes et al. 2018). The newly discovered EPIFUL01 lineage of *Plasmodium* sp. detected in the Checker-throated Stipplethroat is part of a polytomy of diverse *Plasmodium* morphospecies (Fig. 4). Therefore, this new lineage could be another *Plasmodium* species, but further studies on this host are required to confirm. The new *Plasmodium* sp. lineage PIRUB04 found in the Summer Tanager was related to the lineages EMBHER01 in the Sooty-ant Tanager, SETAUD23 in the House Wren, TACTHA01 in the Scarlet Tanager, and PADOM09 in the Bananaquit (evolutionary distances of < 1.7%; Table S1). The latter lineages were related to the *Plasmodium* (*H*.) *cathemerium* morphospecies (ZONCAP15), which was previously documented to infect the Rufous-collared Sparrow (*Zonotrichia capensis*) in Ecuador (Fig. 4). Moreover, the new COLPAS11 lineage of *H*. (*H*.) *paramultipigmentatus* was detected in Common-ground Dove in the Magdalena River Valley. This lineage grouped with the COLPAS03 and COLBUC01 lineages belonging to the morphospecies *H*. (*H*.) *paramultipigmentatus* and *Haemoproteus* (*Haemoproteus*) *multipigmentatus*, respectively (Fig. 4). These lineages are closely associated with birds from the Columbidae family, with species distributed in Mexico, Ecuador, Brazil, Venezuela, and recently reported in Colombia (Valkiūnas et al. 2010; Lotta-Arévalo et al. 2023). Another new lineage, TYRMEL03 of *H*. (*P*.) *tyranni*, was detected in a Tropical Kingbird in the Cauca River Valley. This lineage was associated with the MYMAC03 lineage reported in this study in another individual from the Tropical Kingbird (Fig. 4). The MYMAC03 lineage had previously been detected only through molecular methods in another member of the Tyrannidae family (*Myiodynastes maculatus*) in Brazil (Ferreira et al. 2017). The morphospecies *H.* (*P*.) *tyranni* does not have an associated lineage in the MalAvi database; however, it has a significantly higher number of records in North America (the USA and Canada), while in the rest of the Americas, it has only been previously recorded in Panama, Brazil, and Venezuela, infecting mainly birds of the family Tyrannidae (e.g., *Pitangus sulphuratus* and *T. melancholicus*) (Valkiūnas 2005; Valera et al. 2019; da Silva et al. 2022). Therefore, we consider that the record of *H.* (*P*.) *tyranni* increases its distribution range in South America and provides a lineage associated with this morphospecies. The new lineage SPOMIN01 of *H.* (*P*.) *nucleocentralis* was closely related to the lineage TANDES01 of the same morphospecies (detected only in Brazil). These two lineages have only been reported in two species of the Thraupidae family (*Sporophila minuta* and *Tangara desmaresti*) (Anjos et al. 2021). This study contributes to the knowledge of the diversity of *Plasmodium* and *Haemoproteus* lineages and species present in neotropical resident and migratory birds in tropical lowlands.

### Supplementary Information

Below is the link to the electronic supplementary material.

Supplementary file1 (CSV 105 KB)

## Data Availability

No datasets were generated or analysed during the current study.
